# A Conserved Epitope Mapped with a Monoclonal Antibody against the VP3 Protein of Goose Parvovirus by Using Peptide Screening and Phage Display Approaches

**DOI:** 10.1371/journal.pone.0147361

**Published:** 2016-05-18

**Authors:** Chenxi Li, Hongyu Liu, Jinzhe Li, Dafei Liu, Runze Meng, Qingshan Zhang, Wulin Shaozhou, Xiaofei Bai, Tingting Zhang, Ming Liu, Yun Zhang

**Affiliations:** State Key Laboratory of Veterinary Biotechnology, Harbin Veterinary Research Institute of Chinese Academy of Agricultural Sciences, Harbin, 150001, P. R. China; Universitá Cattolica del S. Cuore, ITALY

## Abstract

**Background:**

Waterfowl parvovirus (WPV) infection causes high mortality and morbidity in both geese (Anser anser) and Muscovy ducks (Cairina moschata), resulting in significant losses to the waterfowl industries. The VP3 protein of WPV is a major structural protein that induces neutralizing antibodies in the waterfowl. However, B-cell epitopes on the VP3 protein of WPV have not been characterized.

**Methods and Results:**

To understand the antigenic determinants of the VP3 protein, we used the monoclonal antibody (mAb) 4A6 to screen a set of eight partially expressed overlapping peptides spanning VP3. Using western blotting and an enzyme-linked immunosorbent assay (ELISA), we localized the VP3 epitope between amino acids (aa) 57 and 112. To identify the essential epitope residues, a phage library displaying 12-mer random peptides was screened with mAb 4A6. Phage clone peptides displayed a consensus sequence of YxRFHxH that mimicked the sequence ^82^Y/FNRFHCH^88^, which corresponded to amino acid residues 82 to 88 of VP3 protein of WPVs. mAb 4A6 binding to biotinylated fragments corresponding to amino acid residues 82 to 88 of the VP3 protein verified that the ^82^FxRFHxH^88^ was the VP3 epitope and that amino acids ^82^F is necessary to retain maximal binding to mAb 4A6. Parvovirus-positive goose and duck sera reacted with the epitope peptide by dot blotting assay, revealing the importance of these amino acids of the epitope in antibody-epitope binding reactivity.

**Conclusions and Significance:**

We identified the motif FxRFHxH as a VP3-specific B-cell epitope that is recognized by the neutralizing mAb 4A6. This finding might be valuable in understanding of the antigenic topology of VP3 of WPV.

## Introduction

Waterfowl parvoviruses (WPVs), including goose parvovirus (GPV) and Muscovy duck parvovirus (MDPV), are widespread in countries that farm waterfowl, where they can cause high morbidity and mortality rates among flocks, leading to considerable economic losses [1, 2]. WPVs are small DNA viruses of the *Parvoviridae* family. Their genomes are approximately 5100 nucleotides in length and contain two open reading frames (ORFs); the right ORF encodes three capsid proteins (VP1, VP2, and VP3), and the left ORF encodes two nonstructural proteins (NS1 and NS2). The C-terminal portion of the VP1 gene contains the coding sequences of VP2 and VP3, which are expressed via differential splicing [3–5]. VP3 is the most variable and abundant of the three core proteins. It induces neutralizing antibodies and confers protective immunity in waterfowls [6,7]. The VP1 polypeptides of GPV and MDPV share 88% identity at the amino acid level [4, 5, 8], which suggests that there may be immunogenic cross reactivity between GPV and MDPV [9].

Although the molecular and biochemical properties of WPVs have been well characterized, less is known about their antigenic structure. Recently, bacterially expressed truncated VP1 proteins were used to identify seven antigenic regions of VP1 that reacted with sera from a GPV-infected goose [10]. However, no epitopes have been identified by using VP3-specific mAbs. By mapping the antigenic structure of a virus, we can identify functional areas involved in recognition, binding, or cell entry. Furthermore, a comprehensive understanding of the antigenic topology of VP3 and characterization of new VP3-specific mAbs would be invaluable in the development of novel VP3-based diagnostic tests or WPV marker vaccines.

In this study, we used Western blotting and a phage-displayed, random 12-mer-peptide library with the neutralizing VP3-specific monoclonal antibody (mAb) 4A6 to map a B-cell epitope on WPV VP3. To our knowledge, this is the first report of an epitope on the VP3 protein of WPV. Its characterization should aid in the development of specific serological diagnosis tests for and vaccines against WPV.

## Materials and Methods

### Ethics Statement

Laboratory animal care and experimentation were performed in accordance with animal ethics guidelines and approved protocols. The Animal Ethics Committee of the Harbin Veterinary Research Institute of the Chinese Academy of Agricultural Sciences approved all animal experiments in this study.

### Virus, anti-GPV/-MDPV goose and duck sera, VP3-specific mAb 4A6, and *In Vitro* Neutralization Assay

GPV EP22 was grown on goose embryo fibroblasts cells (GEF) or embryonated eggs as described previously [11,12]. The anti-GPV and anti-MDPV polyclonal sera were prepared as described previously [12]. The VP3-specific mAb 4A6 was previously developed and characterized [13]. mAb 4A6 neutralizing antibody titers were determined using a virus-based neutralization assay as described previously [9, 12, 14]. Briefly, 100 μL of serially diluted mAb (initial dilution = 1:10 and then 2-fold dilution to 320) was incubated with 100 μL (1×10^2^ TCID_50_) of EP22 for 2 h at 37°C. The virus-mAb mixture (200 μL) was then transferred onto a GEF monolayer in a 96-well plate (triplicate wells). Uninfected healthy mouse serum was diluted in phosphate-buffered saline (PBS) and used as a negative control. Uninfected GEF cells also served as controls. Cytopathic effects (CPE) were observed daily for 7 days; the highest mAb dilutions that could protect >95% cells from CPE were used as the basis for the neutralization titers.

### Broad Epitope Mapping Using Overlapping VP3 Fragments

To localize the epitope on the VP3 protein, we synthesized N- and C-terminally deleted VP3 protein fragments as described previously [12]. Twelve partially overlapping fragments (named P1–P8) spanning VP3 were amplified from pET30a-VP3 and then cloned into the pET-30a vector as described previously [12]. Bam HI/Hind III sites were introduced into the corresponding primers (S1 Table). The recombinant fragments were screened with Western blotting and an ELISA as described previously [15, 16].

### SDS-PAGE and Western Blot

SDS-PAGE and Western blotting were performed as described previously [15, 16]. Briefly, a series of purified truncated VP3 fusion proteins or fragments were mixed with an equal volume of reducing Laemmli sample buffer and electrophoretically separated. Gels were stained with Coomassie brilliant blue or were electroblotted onto nitrocellulose membranes, which were then probed with a 1:1000 dilution of mAb 4A6 before being reacted with horseradish peroxidase (HRP)-conjugated goat anti-mouse immunoglobulin (1:500 dilution) (KPL, MD, USA).

### Epitope Mapping

The epitope was mapped by using the Ph.D-12^TM^ Phage Display Peptide Library Kit (New England BioLabs Inc.) and mAb 4A6 as described previously [16, 17]. The mAb 4A6 was purified from mice ascites fluid by using Protein G Agarose (Invitrogen, Carlsbad, CA, USA) according to manufacturer’s instructions. Three rounds of biopanning were performed. Then, each well of a 96-well plate was coated with 10 μg/mL mAb 4A6 and blocked with blocking buffer. The phage library was then added to the plate and incubated for 1 h. After five washes with TBS buffer, containing increasing concentrations (0.1%, 0.3%, and 0.5%) of Tween-20, 1 M Tris-HCl was added to the plate [16, 17]. The eluted bound phages were then amplified and titered on LB/IPTG/Xgal plates for selection. The ratio of output to input was calculated as the titer of the amplified output phages/the titer of the input phages.

### Phage ELISA and Sequencing of DNA Inserts Displayed by Phage Clones

After the three rounds of biopanning as described above and elsewhere [16, 17], 15 individual phage clones were selected for target binding in the ELISA. Briefly, 96-well plates were coated with 100 ng of mAb 4A6 or mAb anti-porcine IFN-c (Sigma, St Louis, MO, USA) as a negative control. After the coated wells were blocked, the phages (10^10^ pfu/100 μL/well) were added. The coated plates were then washed ten times with TBST, and bound phages reacted with HRP-conjugated sheep anti-M13 antibody (Pharmacia, Piscataway, NY, USA) as described previously [16, 17]. Color development was achieved by adding substrate solution containing o-phenylenediamine (OPD). The positive phage clones were sequenced with sequencing primer as described previously [16].

### Sequence Analysis

To assess the level of conservation of the epitope among WPVs, we assessed the sequence alignment of the epitope and the corresponding locations in VP3 of eleven GPV and two MDPV strains using the DNASTAR Lasergene program (DNASTAR Inc., Madison, WI, USA)[18].

### Identification of the Essential Amino Acids in the Epitope

To precisely define the epitope, six biotinylated peptides YNRFHCH, FNRFHCH, NRFHCH, RFHCH, RFHAH, and RFHQH spanning 82–88 amino acids of VP3 were synthesized (with purity >95%) by GenScript China Inc.,. Peptides reactivities were analyzed by ELISA as described above. Peptide YIRTPACWD and VP3 protein were used as negative and positive control, respectively.

### Epitope Peptide Reactivity to Anti-GPV/-MDPV Goose and Duck sera

Dot blotting was performed by spotting a synthesized epitope peptide solution onto a nitrocellulose membrane as described previously [16, 17]. Briefly, Approximately 1 μg of synthesized peptide YNRFHCH or an unrelated negative control peptide (YIRTPACWD from duck reovirus σB protein [15]), and positive control full length VP3 fusion protein of GPV [12] diluted with TNE buffer was spotted onto the nitrocellulose membrane. The membrane was then incubated with anti-GPV/-MDPV goose or duck sera (diluted 1:200 in PBS) and probed with a 1:500 dilution of HRP-conjugated goat anti-duck IgG (KPL, MD, USA) at 37°C for 1 h, respectively.

## Results

### Neutralization Titer of mAb 4A6

The neutralizing activity of mAb 4A6 was determined by using an *in vitro* neutralization assay with GEF; mAb 4A6 neutralized GPV with a neutralization titer (NT_50_) of 80.

### Approximate Localization of the VP3 Epitope

**Fig 1:**
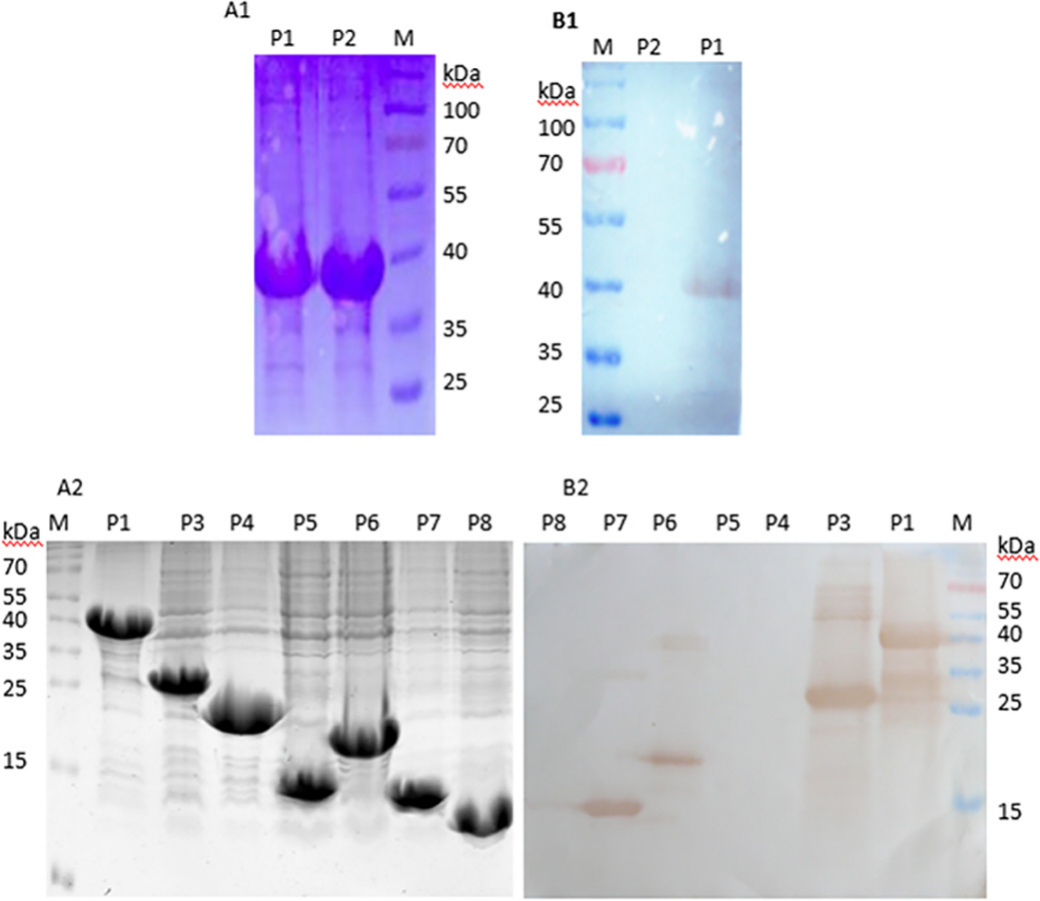
**SDS-PAGE (A1 and A2) and Western blot analysis (B1 and B2) of truncated VP3 protein.** Reactions of peptides P1 and P2 with mAb 4A6 (B1); reactions of peptides P1 and P3–P8 (B2) with mAb 4A6 (B2); lane M, protein molecular marker.

**Fig 2:**
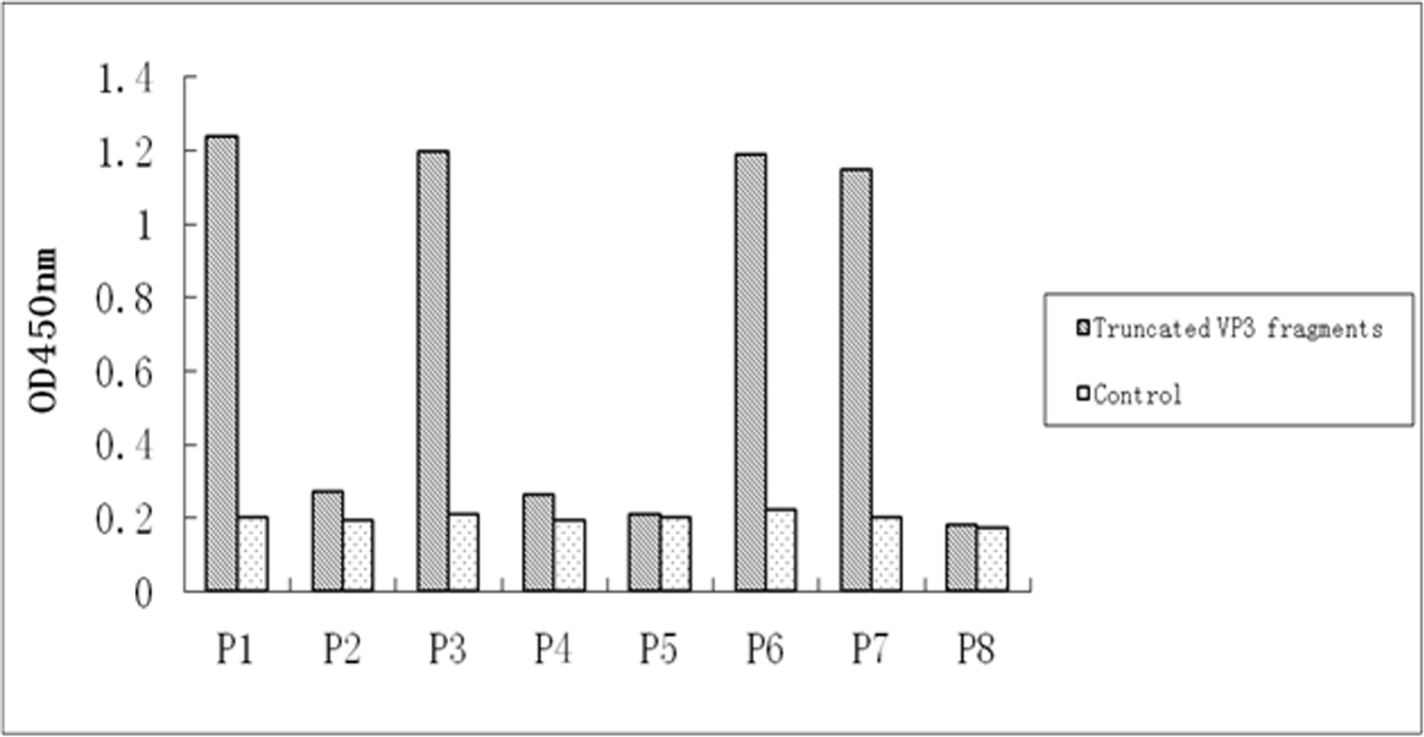
Identification of the general antigenic region of VP3 by ELISA. Truncated fragments P1, P3, P6, and P7 reacted with mAb 4A6. There was no cross-reactivity with the pET30a control.

Western blotting of truncated fragments of VP3 showed that P1 (aa 1–271) was recognized by mAb 4A6 (Fig 1A1 and Fig 1B1) but that P2 (aa 271–535) was not, suggesting that mAb 4A6 recognized the antigenic region in the amino half of the VP3 protein. To further localize this epitope, we divided the 1–271 aa region into six partially overlapping fragments. The screening results showed that P1 (aa 1–271), P3 (aa 1–140), P6 (aa 57–132), and P7 (aa 57–112) were all detected by mAb 4A6 (Fig 1A2 and 1B2), whereas P4 (aa 127–271), P5 (aa 1–56), and P8 (aa 15–29) were not, indicating that mAb 4A6 recognizes an antigenic domain within amino acids 57–112. The ELISA results (Fig 2) were consistent with the Western blotting data.

### Epitope Prediction

**Fig 3:**
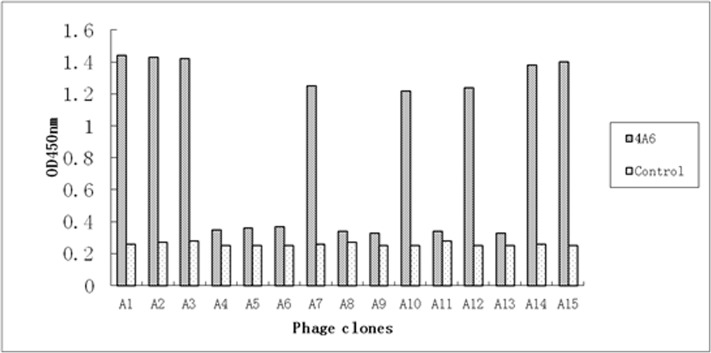
Detection of selected phages for antibody binding by Phage ELISA. Fifteen selected phage clones after three rounds of biopanning were detected by mAb 4A6 or by the anti-porcine IFN-c mAb (negative control).

To map the precise location of the VP3 epitope, we screened a phage displayed 12-mer random peptide library using mAb 4A6. After three rounds of biopanning, we selected 15 phage clones and evaluated their reactivity with mAb 4A6 and with the negative control anti-porcine IFN-c mAb. Eight clones (A1–A3, A7, A10, A12, A14, and A15) reacted with mAb 4A6 (OD450 nm, ≥1.20) but did not react with the anti-porcine IFN-c mAb (OD450 nm, ˂0.27) (Fig 3). The other 7 clones (A4–A6, A8, A9, A11, and A13) were less reactive with mAb 4A6 (OD450 nm, OD˂0.36). When we sequenced the eight phage clones with the high OD values, we found the consensus sequence YxRFHxH (Table 1). Sequence alignment showed that this sequence is quite similar to the VP3 region 79 to 88 (^79^YFDFNRFHCH^88^) of GPV strain EP22, but ^79^Y seems too far from the other putative binding residues for inclusion in the linear epitope. A more reasonable interpretation might be that the Tyr is selected as an alternative to the Phe at position 82 or perhaps the library itself was biased toward Tyr. It is necessary to decide whether ^82^F should be included in this epitope.

**Table 1:** Peptide sequences of eight selected phage clones.

Phage clone	Phage Sequence															
A1			D	P	Q	Y	T	R	F	H	Q	H	P	Q		
A2			D	Q	H	Y	T	R	F	H	Q	H	F	R		
A3		Q	L	G	H	Y	D	R	F	H	K	H	P			
A7	S	M	N	P	T	W	L	R	F	H	P	H				
A10			Y	P	T	F	E	R	F	H	T	H	T	P		
A12			S	S	M	L	N	R	F	H	I	H	T	L		
A14				I	P	Y	T	R	F	H	D	H	Q	Y	T	
A15	S	T	S	A	S	Y	T	R	F	H	S	H				
Consensus						Y		R	F	H		H				
Virus EP22			Y	F	D	Y/F	N	R	F	H	C	H	F	S	P	R

### Precision Mapping of the Epitope

**Fig 4:**
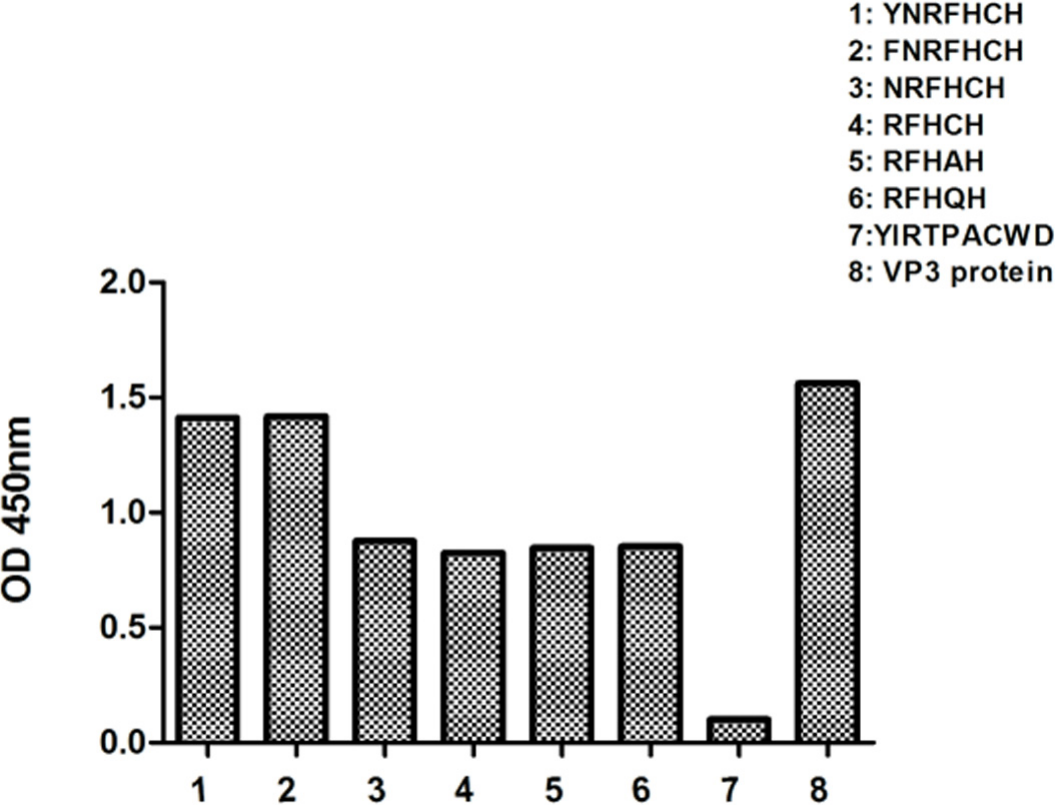
The influence of synthesized peptides on binding to mAb 4A6 detected by ELISA. Three independent assays were performed for each selected peptides.

The influence of ^82^Y or ^82^F or ^87^C on binding to mAb 4A6 was tested by ELISA. ELISA results showed that the peptide YNRFHCH and FNRFHCH displayed a 1.5-times-higher reactivity with 4A6 than the peptide NRFHCH, suggesting that ^82^Y or ^82^F is important in this epitope. RFHCH, RFHAH, and RFHQH showed similar reactivity to mAb 4A6, indicated that ^87^C is replaceable in this epitope binding reactivity (Fig 4). Negative control peptide (YIRTPACWD) did not show any reaction with mAb 4A6. These experimental observations strongly suggested that the ^82^F was important amino acid residue for binding to mAb 4A6 and the ^82^FxRFHxH^88^ could be a B-cell epitope of the VP3 protein of GPV.

#### Immunological reaction of epitope to goose/duck anti-WPV sera

**Fig 5:**
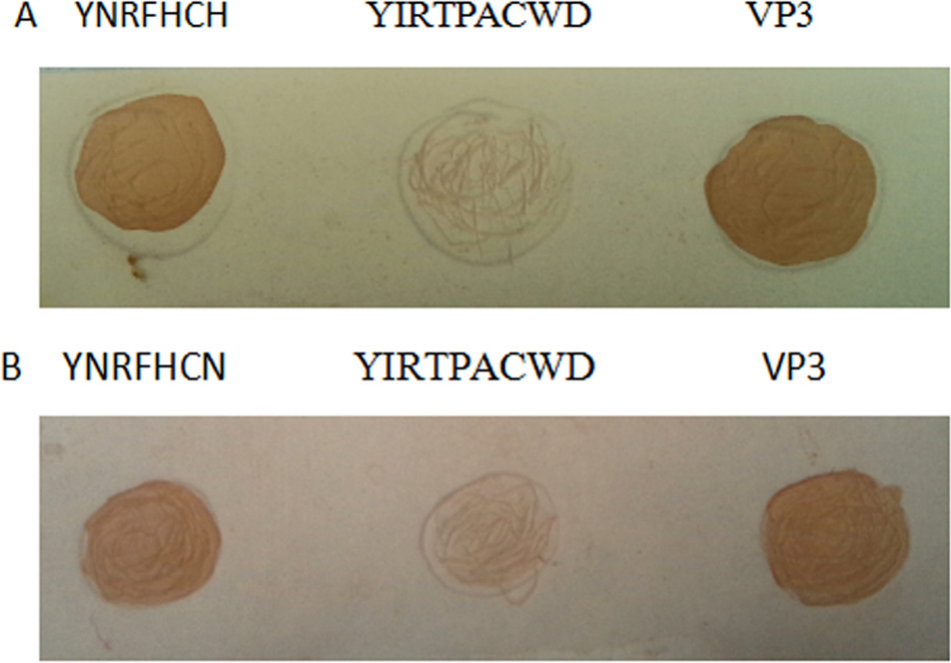
**Dot blotting assay of anti-GPV/-MDPV goose (A) and duck (B) sera to epitope peptide YNRFHCH.** YIRTPACWD and VP3 protein were used as negative and positive control, respectively.

Dot blotting assay was used to test whether the identified motif FNRFHCH recognized by goose/duck anti-WPV sera. Dot blot analysis showed that the peptide FNRFHCH and VP3 protein were recognized by both goose and duck anti-WPV sera (Fig 5), but did not react with healthy goose/duck sera. Negative control peptide (YIRTPACWD) did not show any reaction to goose/duck anti-WPV sera, indicating that the motif represented a B-cell epitope of the VP3 protein of WPV.

#### Epitope FxRFHxH is highly conserved among GPV and MDPV strains

**Fig 6:**
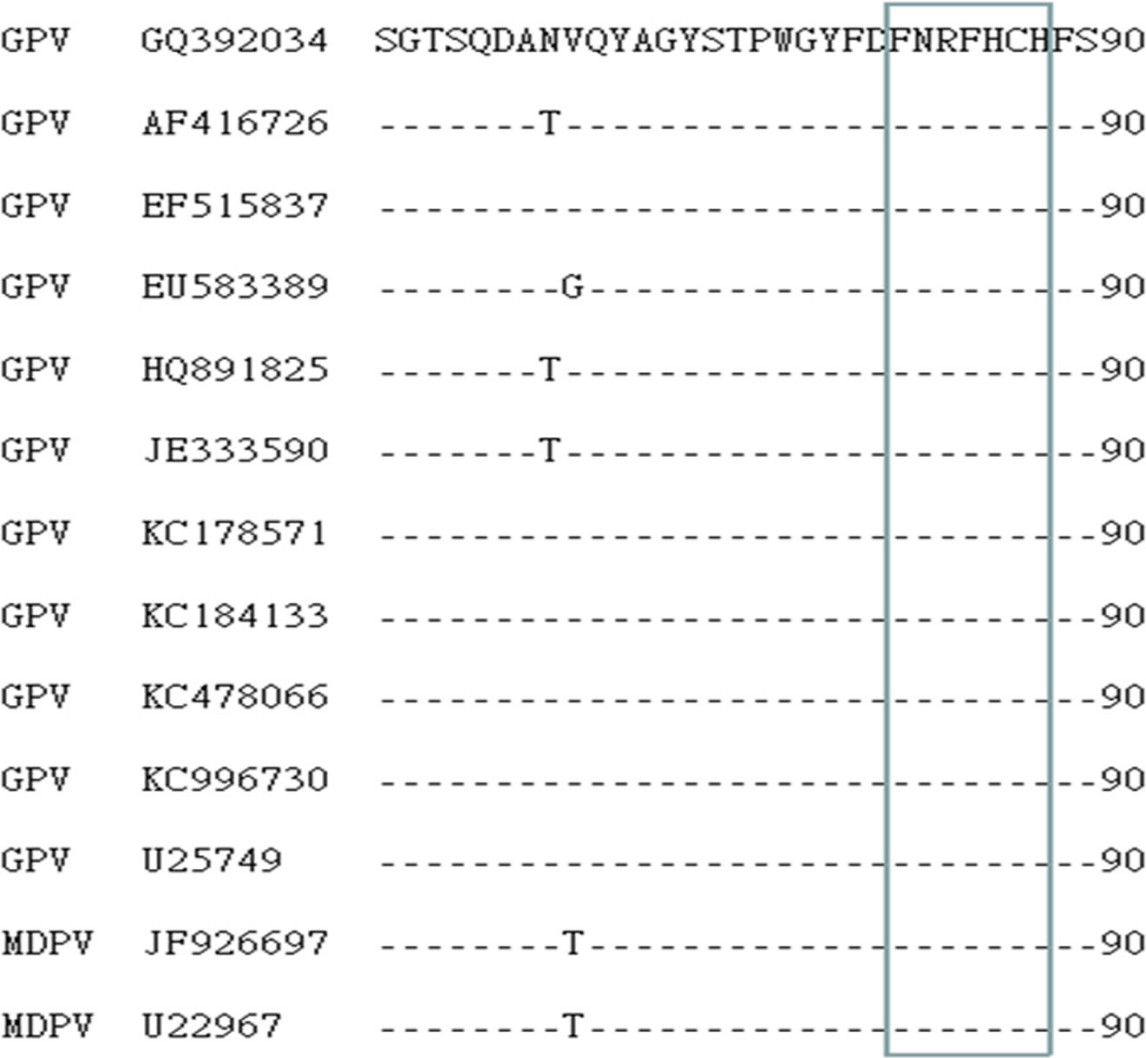
Sequence alignment of the epitope-coding region in the VP3 protein of 13 WPV strains. Amino acid positions for each sequence are numbered on the right. The sequence for the GPV EP22 strain is shown at the top; the dashes indicate identical amino acids. The identified epitope region is boxed in red.

To determine whether the FxRFHxH epitope is conserved in the VP3 protein of WPV, we aligned the VP3 partial sequence, including the epitope region identified in this study, with other GPV and MDPV sequences available in GenBank (Table 2). This sequence alignment revealed that all amino acids in the motif region were identical between the available GPV and MDPV strains (Fig 6), indicating that this motif represents a conserved epitope in the VP3 protein of GPV and MDPV.

**Table 2:** Virus strains used in the sequence analysis in this study.

Species	Strain	Accession no.	Site of Isolation
GPV	GPV EP2	GQ392034	China
GPV	GPV-YG	AF416726	China
GPV	DY	EF515837	China
GPV	82-0321V	EU583389	China
GPV	GDaGPV	HQ891825	Taiwan
GPV	SH	JF333590	China
GPV	Y	KC178571	China
GPV	E	KC184133	China
GPV	SHFX1201	KC478066	China
GPV	YZ99-6	KC996730	China
GPV	B	U25749	Hungary
MDPV	P	JF926697	China
MDPV	FM	U22967	Hungary

## Discussion

Monoclonal antibodies with well-defined epitopes provide an experimental platform for studying antigen structure and developing diagnostic reagents and epitope vaccines [19,20]. The protein VP3 is able to induce neutralizing antibodies and is also confirmed to be a major candidate antigen for the development of vaccines and serologic diagnostic tests [7,12, 21]. The precise definition of the epitopes in VP3 is important not only for understanding the mechanism of VP3-mediated protection but would also contribute to developing epitope-based marker vaccines. Antibody detection assays based on whole antigens with multiple epitopes show greater sensitivity, but cross-reactions are often observed. Epitopes or mimics of natural antigenic determinants, which mainly originate from dominant responses, favor more highly reactive antigens due to their optimized structure or functional properties. They should therefore be more applicable in serologic diagnostic tests [22].

Phage-display and peptide screening have been widely used and offer an attractive approach for the identification of epitopes for monoclonal antibodies. In this investigation, we identified a conserved neutralizing epitope on VP3 of GPV by using both phage display and peptide screening methods with a VP3-specific neutralizing mAb 4A6. Peptide screening with overlapping VP3 fragments mapped a general antigenic domain between amino acids 57 and 112. To confirm this finding, a ph.D-12^TM^ Phage Display Peptide Library was used to map the epitope location on VP3. Phage ELISA and sequencing alignment results showed that YxRFHxH might be the epitope.

To confirm the essential amino acid residues (amino acids ^82^Y and ^87^C, ^82^FNRFHCH^88^) in this epitope region, six designed peptides spanning 82–88 of this epitope coupled with ELISA analysis demonstrated that motif FxRFHxH is required for mAb 4A6 recognition. Tyr is selected as an alternative to the Phe at position 82 might be that the library itself was biased toward Tyr. This motif is identical to the sequence ^82^FNRFHCH^88^ of the VP3 protein of WPV. Thus, the peptide FxRFHxH is the minimum motif of the epitope needed to retain maximal binding to mAb 4A6. The peptide was also recognized by WPV positive duck/goose sera, revealing the importance of the seven amino acids of the epitope in antibody-epitope binding reactivity. Sequence alignments of eleven GPV and two MDPV strains demonstrated that the motif was highly conserved among WPV, indicating that it is a broad group-specific epitope.

### Conclusions

In summary, we identified a conserved neutralizing B-cell epitope on the VP3 protein of WPV. This epitope may have value in understanding of the antigenic topology of VP3 of WPV.

## Supporting Information

S1 Table Primers for truncated VP3 protein fragments. (TIF)
