# Supplementary figures and images for: A Conserved Epitope Mapped with a Monoclonal Antibody against the VP3 Protein of Goose Parvovirus by Using Peptide Screening and Phage Display Approaches
Source: PLoS One. 2016 May 18;11(5):e0147361. doi: 10.1371/journal.pone.0147361 (PMC4871417; doi:10.1371/journal.pone.0147361)

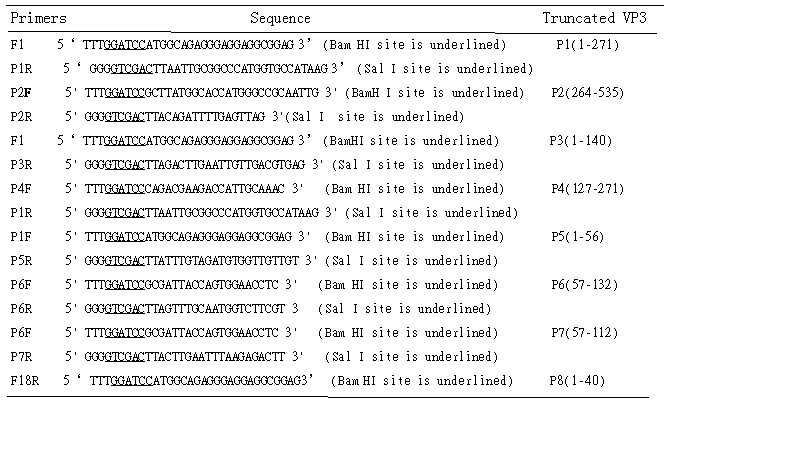

Supplement: S1 Table — (TIF) [file pone.0147361.s001.tif]
